# Acupuncture at local and distant points for tinnitus: study protocol for a randomized controlled trial

**DOI:** 10.1186/1745-6215-13-224

**Published:** 2012-11-23

**Authors:** Guang-Xia Shi, Li-Li Han, Li-Ying Liu, Qian-Qian Li, Cun-Zhi Liu, Lin-Peng Wang

**Affiliations:** 1 Acupuncture and Moxibustion Department, Beijing Hospital of Traditional Chinese Medicine affiliated to Capital Medical University, 23 Meishuguanhou Street, Dongcheng District, Beijing, 100010, China

## Abstract

**Background:**

Tinnitus is the perception of a sound in the absence of an objective physical source. Up to now, there is no generally accepted view how these phantom sounds come about, and also no efficient treatment. Patients are turning to complementary or alternative medical therapies, such as acupuncture. Based on the theory of traditional Chinese medicine, acupoints located on both the adjacent and distal area of the disease can be needled to treat disease. Furthermore, the way of combining acupoints is for strengthening the curative effect. We aim to evaluate the efficacy of acupuncture at local points in combination with distal points in subjective tinnitus patients.

**Method:**

This trial is a randomized, single-blind, controlled study. A total of 112 participants will be randomly assigned to one of four treatment groups receiving acupuncture treatment for 4 weeks. The primary outcome measure is subjective tinnitus loudness and annoyance perception, which is graded using the Visual Analogue Scale (VAS). The assessment is at baseline (before treatment initiation), 4 weeks after the first acupuncture session, and 8 weeks after the first acupuncture session.

**Discussion:**

Completion of this trial will help to identify whether acupuncture at local acupoints in combination with distal acupoints may be more effective than needling points separately.

**Trial registration:**

International Standard Randomized Controlled Trial Number Register: ISRCTN29230777

## Background

### Tinnitus

Subjective tinnitus is a frequent auditory sensation (for example, a tone, hissing, or buzzing sound, and sometimes combinations of such perceptions) experienced in the absence of an external or internal acoustic stimulus. An estimated prevalence in the adult population across studies is about 10% to 15% [1-3], where 1% to 3% of the population has severe, distressing tinnitus. Among severe sufferers, tinnitus causes disability associated with concentration deficits, hypersensitivity to sounds, anxiety, depression, irritability, agitation, and insomnia. Often a combination of several complaints could disrupt daily activities and has a negative impact on quality of life. It represents a worldwide major healthcare problem with an enormous social and economic demand for therapeutic treatment [4,5]. Pharmacotherapy (antidepressants, benzodiazepines), cognitive therapies, or electronic devices that try to cancel the tinnitus have all been tried either separately or in combination but the success rate is not high. Attempts to develop evidence-based therapies have been thwarted by a poor understanding of the pathophysiology [6,7]. Increasingly, patients are turning to complementary and alternative medical therapies. Acupuncture is among the most popular.

### Acupuncture

Acupuncture has been used to treat tinnitus for a long time. Neuroscience studies related the effects of acupuncture to neuronal stimulus, activation of endogenous opioid mechanisms and neuropeptides which stimulate specific brain structures [8]. It has been reported that acupuncture can yield immediate relief, both from the loudness and the disturbing quality of tinnitus, significant improvement in quality of life, less tension, and better sleep [9-12]. In contrast, an analysis of six randomized clinical trials of acupuncture for tinnitus failed to demonstrate any efficacy [13]. Based on the theory of traditional Chinese medicine (TCM), acupoints located on both the adjacent and distal area of the disease can be needled to treat disease. Furthermore, the way of combining these acupoints is often used for strengthening the curative effect.

### Aims

The aim of our study is to evaluate the efficacy of acupuncture at local points in combination with distal points in subjective tinnitus patients.

## Methods

We perform the study according to common guidelines for clinical trials (Declaration of Helsinki, International Conference on Harmonisation (ICH)/WHO Good Clinical Practice standards (GCP) including certification by an external audit). The trial protocol has been approved by the Research Ethical Committee of Beijing Hospital of Traditional Chinese Medicine Affiliated to Capital Medical University. This trial was registered with ISR CTN at Current Controlled Trials (ISRCTN29230777).

### Population

Patients will be recruited in acupuncture clinic, Beijing Hospital of Traditional Chinese Medicine affiliated to Capital Medical University with a target sample size of 112 subjects. The trial is executed from February 2012 to December 2014.

### Inclusion criteria

Patients who require for all of the following conditions will be considered for enrollment:

1. Diagnosis of subjective tinnitus, unilateral or bilateral

2. Age 18 to 65 years, either sex

3. Tinnitus duration of at least 3 months

4. Not to receive another treatment during the clinical trial period

5. Written and informed consent

### Exclusion criteria

1. Objective tinnitus (objective tinnitus is audible to the examining/auscultating physician, whereas subjective tinnitus can only be perceived by the patient)

2. Underlying disease or history:

2.1. Cerebral vascular events

2.2. Neurodegenerative disorders

2.3. Otitis media

2.4. Acoustic tumor

2.5. Prior brain surgery

2.6. Inner ear malformation

2.7. Head trauma

2.8. Ototoxic drug medication

3. Women in pregnancy and lactation or without contraception

4. Inability to correct use of test equipment: unable to cooperate during audiologic examination

5. Patients who cannot communicate reliably with the investigator or who are not likely to cope with the requirements of the trial

### Interventions

This trial is a randomized, single-blind, controlled study. Participants will receive acupuncture for 4 weeks. All candidates go through a standardized interview and receive more information about the study and the treatments. They also undergo audiological testing of hearing thresholds, minimal masking levels, and loudness discomfort levels carried out by an audiologist in Tongren Hospital affiliated to Capital Medical University.

**Figure 1:**
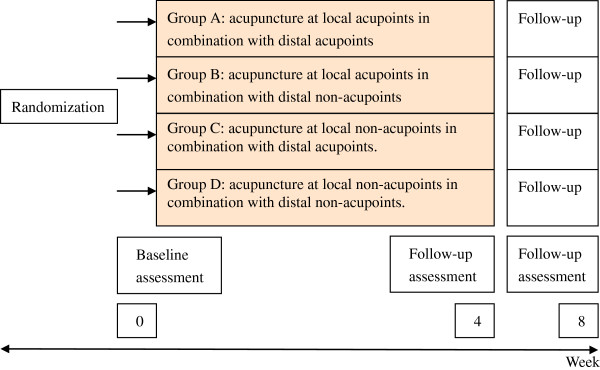
Flowchart.

Time points are as follows (Figure 1):

Visit 1: screening

Visit 2: treatment initiation, participants will receive acupuncture for 4 weeks

Visit 3: 4 weeks after first acupuncture, follow-up and treatment finish

Visit 4: 8 weeks after first acupuncture, follow-up

**Figure 2:**
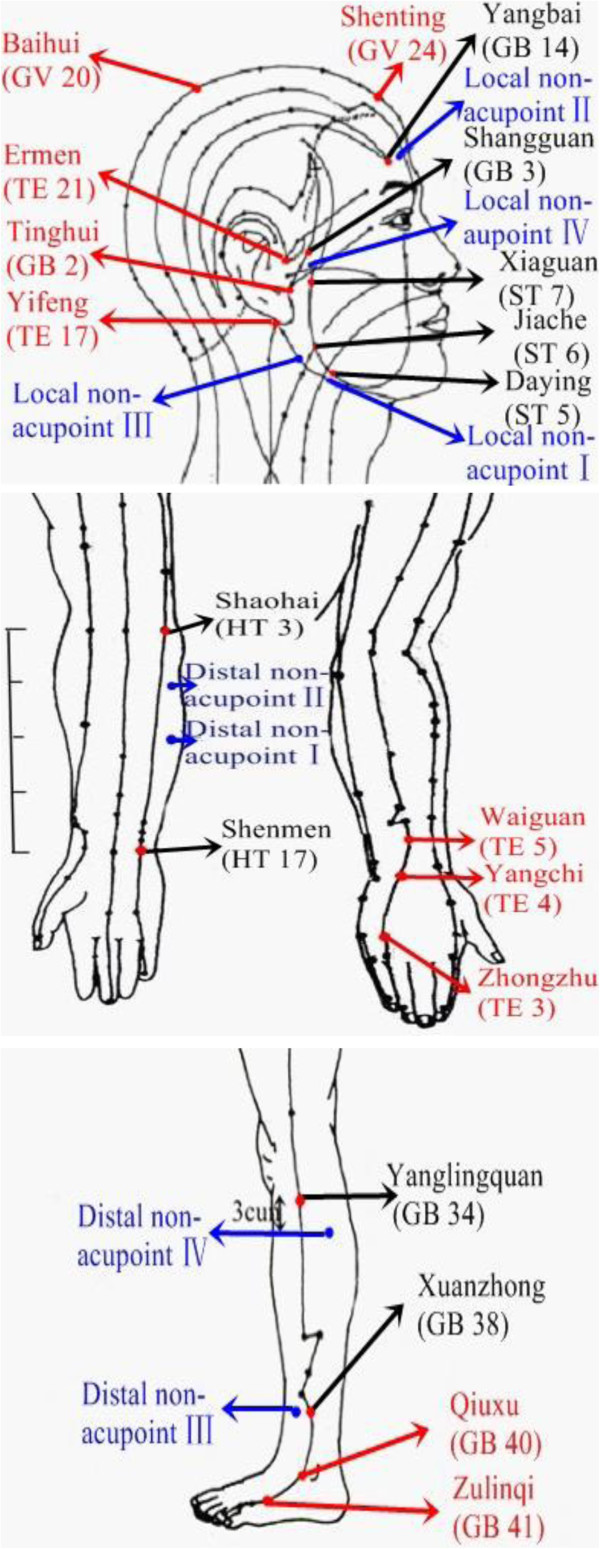
The points used in the trial.

Patients who meet the inclusion criteria and none of the exclusion criteria are randomized to one of four treatment groups: group A will receive acupuncture at local acupoints in combination with distal acupoints; group B will receive acupuncture at local acupoints in combination with distal non-acupoints; group C will receive acupuncture at local non-acupoints in combination with distal acupoints; group D will receive acupuncture at local non-acupoints in combination with distal non-acupoints. Acupuncture points selected as follows (Figure 2):

 Local acupoints: Baihui (GV 20), Shenting (GV 24), Yifeng (TE 17), Tinghui(GB 2), Ermen (TE 21)

 Distal acupoints: Waiguan (TE 5), Zhongzhu (TE 3), Qiuxu (GB 40), Zulinqi (GB 41)

 Distal non-acupoints

Distal non-acupoint I: the radial side of forearm, the ulner side of the middle of the line joining Shaohai (HT 3) and Shenmen (HT 7), between heart meridian of hand-shaoyin and small intestine meridian of hand-taiyang

Distal non-acupoint II: the radial side of forearm, the junction of the upper 1/4 and lower 3/4 of the line joining HT 3 and HT 7, between heart meridian of hand-shaoyin and small intestine meridian of hand-taiyang

Distal non-acupoint III: lateral side of lower leg, 3 cun above the tip of external malleolus, 1.5 cun behind anterior crest of the tibia, between the stomach meridian of foot-yangming and gall bladder meridian of foot-shaoyang

Distal non-acupoint IV: lateral to the shank, 3 cun below Yanglingquan (GB34), the midway between gall bladder meridian of foot-shaoyang and bladder meridian of foot-taiyang

 Local non-acupoints

Local non-acupoint I: one finger-breadth (middle finger) inferior to Daying (ST5) in the body of mandible

Local non-acupoint II: midpoint between Yangbai (GB14) and the midline of the head, between governor meridian and gall bladder meridian of foot-shaoyang

Local non-acupoint III: high spot of the angle of mandible

Local non-aupoint IV: on zygomatic arch, the midway between ST 7 and GB 3

Acupuncture will be performed with sterile needles by one therapist with more than 22 years of experience and an acupuncture license from the Chinese medicine practitioner license from the Ministry of Health of the People’s Republic of China. Treatment will be conducted over a period of 4 weeks, at a frequency of two sessions per week. No additional treatment is allowed.

### Randomization and blinding

A research coordinator screen and enroll participants at clinic. After participants completed a baseline evaluation, another research coordinator who is uninvolved with data collection randomly assigns them to one of four treatment groups by using a computer-generated, blocked random-allocation sequence. The random list is generated with SAS software (SAS Institute, Inc., Cary, NC, USA). This research coordinator informs the acupuncturist of the treatment assignment.

The patients, data collection staff, and data analysts are blinded during the study period. The acupuncturist is not blinded to the treatments they delivered because acupuncture manipulation makes it impossible. During the intervention, acupuncturist and the personnel who collect data are segregated immediately after the treatment start and are instructed not to exchange information with each other.

### Primary outcome measures

Subjective tinnitus loudness and annoyance perception is graded using the VAS. The assessment is at baseline (before treatment initiation), 4 weeks after the first acupuncture session, and 8 weeks after the first acupuncture session.

### Secondary outcome measures

 Change of tinnitus severity according to the tinnitusquestionnaire of Tinnitus Handicap Inventory (THI):

1.1. F: Functional subscale (11 factors)

1.2. E: Emotional subscale (9 factors)

1.3. C: Catastrophic subscale (5 factors)

 Changes of tone and noise parameters of tinnitus are assessed by tinnitus matching (screening *versus* week 8).

 Perceived credibility of acupuncture would be evaluated by the Treatment Credibility Scale (TCS) after a 4-week acupuncture session. It is a five-item questionnaire ranging from 1 (not at all) to 5 (very confident); items are averaged to provide a single treatment credibility score, with high scores reflecting high treatment credibility.

 To evaluate the adequacy of blinding, we will ask participants to rate how certain they are that they have received traditional acupuncture or new method of acupuncture on a seven-point scale (1 very sure, 7 very uncertain) after 4 weeks of treatment.

Each question of the THI can be answered by the patient with either ‘often’ (4 points), ‘sometimes’ (2 points), or ‘never’ (0 points) with a maximum total score of 100 points indicating most severe suffering from tinnitus. The assessment is at baseline (before treatment initiation), 4 weeks after the first acupuncture, and 8 weeks after the first acupuncture.

Participants should report the adverse events they experienced, including discomfort or bruising at the sites of needle insertion, nausea, or feeling faint after each treatment.

### Statistical methods

The trial aims to detect a difference in loudness and annoyance perception of tinnitus between the four study groups. This is deemed a clinically significant difference of average 20-point reduction on the VAS scores of loudness in acupuncture group when comparing with the sham-control group based on previously published studies [14]. The standard deviation is 25.9 in acupuncture group and 23.8 in the sham-control group, respectively, as previous studies have suggested [15].

The following formula was used for a four-group trial [16]:

(1) $$n1=n2=2{\left[\frac{\left(\mathrm{u\alpha}+\mathrm{u\beta}\right)s}{\delta }\right]}^{2}$$

Calculations are performed using 80% power, a 5% significance level (two-side). The required sample size is approximately 24 participants for each group. We plan to enroll a total of 112 participants with 28 in each group, allowing for a 25% withdrawal rate.

### Data analysis

Data will be analyzed according to the 2×2 randomized factorial study designs. The statistical model includes two fixed factors (both local acupoints and distal acupoints). Interaction is evaluated using the interaction term in the ANOVA model and by visual assessment of profile plots. As no interaction between local acupoints and distal acupoints is detected, results are presented as a factorial design (for example, all patients receiving acupuncture at local acupoints in combination with distal non-acupoints; and all patients receiving acupuncture at local non-acupoints in combination with distal acupoints).

Data analysis will be conducted by statisticians who are independent from the research team. Every analysis is conducted using the SPSS software (SPSS 12.0 KO for Windows ©).

## Discussion

We have presented the design and the protocol for the randomized controlled trial of acupuncture at local and distant points for subjective tinnitus. Completion of this trial will help to identify whether acupuncture at local acupoints in combination with distal acupoints may be more effective than needling points separately.

It is hoped that this study will encourage renewed interest in acupuncture’s potential for treating people with tinnitus. Recent researches have created a climate where acupuncture might be assumed to be ineffective [17]. However, there is as yet inadequate evidence on which to base a judgment on acupuncture’s effectiveness for tinnitus. Well-designed randomized controlled trials where patients are representative of people with tinnitus in the general population are needed.

In this study, the side-effects of acupuncture will be reported by patients. Several large surveys have also provided evidence that acupuncture is a relatively safe treatment [18]. The results could also provide consumers with information about viable choices. If acupuncture is demonstrated to be effective and safety in tinnitus, there may be a number of patients with these conditions who seek out acupuncture rather than pharmacotherapy.

Patients are informed in a manner suggesting that two different types of acupuncture treatment were compared. One type is traditional, another is new, and the effect of both is uncertain, not mentioning terms such as ‘placebo’ or ‘sham’. Similar strategies of informed consent have been used in most previous acupuncture trials [19].

However, our study has several limitations. Deciding on an appropriate control procedure for clinical studies on acupuncture is a particular challenge [20]. Previous randomized, controlled trials of acupuncture suggest that needling of acupoints is as effective as non-points, in particular for pain releif, although both interventions were more effective than a waiting list control [21-23]. Few guidelines exist, however, for identifying appropriate sham point locations; the depth, direction, and duration of needle insertion; or the need for needle stimulation. In the present study, physicians were instructed to avoid manual stimulation of the needles and provocation of Deqi at non-acupuncture points. However, we cannot rule out that this intervention may have some physiological effects.

One limitation concerns the fact that this is a single-center study. It does not lend the results to great generalizability to more diverse sets of patients in more diverse settings. Our directed acupuncture treatment is uniformly applied, and adjunctive therapies are not used. In addition, acupuncturists in diverse settings with different philosophies, backgrounds, training, and clinical experiences might have chosen different technique for the directed acupuncture intervention. Therefore, the external validity can be somewhat hard to achieve. But we ensure that the risk of contamination between groups will be minimized, while maintaining the integrity of the experimental group comparison. As a result, whether the findings can be generalized to the clinical setting is unclear and more work is needed.

Another limitation is that the therapist is not blinded in the present trial. A blinded study of acupuncture is challenging to conduct because it is almost impossible to blind acupuncturists to the treatments they are delivering. This study does not allow us to determine whether the observed effectiveness is due to placebo effects, intensity of provider contact, or a physiologic effect of needling. Therefore, a bias due to unblinding cannot be ruled out. In addition, the sample size in the present study is relatively small.

## Trial status

The trial is currently in recruitment phase.

## Competing interests

The authors declare that they have no competing interests.

## Authors’ contributions

CZL conceived of the study and prepared the initial protocol. LPW made amendments and participated in design of the trial protocol. GXS drafted the manuscript and participated in design of the study. LLH participated in the development of the acupuncture point protocol. QQL and LYL participated in the design of the study. All authors read and approved the final manuscript.
